# Salivary miRNA profiles identify children with autism spectrum disorder, correlate with adaptive behavior, and implicate ASD candidate genes involved in neurodevelopment

**DOI:** 10.1186/s12887-016-0586-x

**Published:** 2016-04-22

**Authors:** Steven D. Hicks, Cherry Ignacio, Karen Gentile, Frank A. Middleton

**Affiliations:** Department of Pediatrics, Milton S. Hershey Medical Center of Penn State University, Hershey, PA USA; Partek Incorporated, St. Louis, MO USA; Department of Neuroscience & Physiology, State University of New York, Upstate Medical University, 750 East Adams Street, Syracuse, NY 13210 USA; Department of Psychiatry & Behavioral Sciences, State University of New York, Upstate Medical University, Syracuse, NY USA; Department of Biochemistry & Molecular Biology, State University of New York, Upstate Medical University, Syracuse, NY USA

**Keywords:** miRNA, Next generation sequencing, RNA-Seq, Biomarker, Saliva

## Abstract

**Background:**

Autism spectrum disorder (ASD) is a common neurodevelopmental disorder that lacks adequate screening tools, often delaying diagnosis and therapeutic interventions. Despite a substantial genetic component, no single gene variant accounts for >1 % of ASD incidence. Epigenetic mechanisms that include microRNAs (miRNAs) may contribute to the ASD phenotype by altering networks of neurodevelopmental genes. The extracellular availability of miRNAs allows for painless, noninvasive collection from biofluids. In this study, we investigated the potential for saliva-based miRNAs to serve as diagnostic screening tools and evaluated their potential functional importance.

**Methods:**

Salivary miRNA was purified from 24 ASD subjects and 21 age- and gender-matched control subjects. The ASD group included individuals with mild ASD (DSM-5 criteria and Autism Diagnostic Observation Schedule) and no history of neurologic disorder, pre-term birth, or known chromosomal abnormality. All subjects completed a thorough neurodevelopmental assessment with the Vineland Adaptive Behavior Scales at the time of saliva collection. A total of 246 miRNAs were detected and quantified in at least half the samples by RNA-Seq and used to perform between-group comparisons with non-parametric testing, multivariate logistic regression and classification analyses, as well as Monte-Carlo Cross-Validation (MCCV). The top miRNAs were examined for correlations with measures of adaptive behavior. Functional enrichment analysis of the highest confidence mRNA targets of the top differentially expressed miRNAs was performed using the Database for Annotation, Visualization, and Integrated Discovery (DAVID), as well as the Simons Foundation Autism Database (AutDB) of ASD candidate genes.

**Results:**

Fourteen miRNAs were differentially expressed in ASD subjects compared to controls (*p* <0.05; FDR <0.15) and showed more than 95 % accuracy at distinguishing subject groups in the best-fit logistic regression model. MCCV revealed an average ROC-AUC value of 0.92 across 100 simulations, further supporting the robustness of the findings. Most of the 14 miRNAs showed significant correlations with Vineland neurodevelopmental scores. Functional enrichment analysis detected significant over-representation of target gene clusters related to transcriptional activation, neuronal development, and AutDB genes.

**Conclusion:**

Measurement of salivary miRNA in this pilot study of subjects with mild ASD demonstrated differential expression of 14 miRNAs that are expressed in the developing brain, impact mRNAs related to brain development, and correlate with neurodevelopmental measures of adaptive behavior. These miRNAs have high specificity and cross-validated utility as a potential screening tool for ASD.

**Electronic supplementary material:**

The online version of this article (doi:10.1186/s12887-016-0586-x) contains supplementary material, which is available to authorized users.

## Background

Autism spectrum disorder (ASD) is a continuum of neurodevelopmental characteristics that includes deficits in communication and social interaction, as well as restrictive, repetitive interests and behaviors. Pediatricians have an opportunity to improve outcomes for children with ASD through early diagnosis and referral for evidence-based behavioral therapy. Unfortunately, the first sign of ASD commonly recognized by pediatricians is a deficit in communication and language that does not manifest until 18–24 months of age [1]. Current screening tools for ASD in this age group include the Infant Toddler Checklist (ITC; also known as the Communication and Symbolic Behavior Scales and Developmental Profile) and the Modified Checklist for Autism in Toddlers-Revised (M-CHAT-R). The ITC may be used to identify developmental deficits in children ages 9–24 months, but has limited utility in distinguishing basic communication delays from overt ASD [2]. The M-CHAT-R may be employed between 16 and 30 months. It requires a follow-up questionnaire for positive screens, which occur in approximately 10 % of children. Thus, the mean age of diagnosis for children with ASD is 3 years, and approximately half of these are false-positives [3].

Biomarker screening, which can be performed anytime after birth, represents an attractive addition to the ASD screening toolkit. A significant genetic component exists in ASD. ASD concordance rates are 50–90 % among monozygotic twins compared with 0–30 % among dizygotic twins [4], while full siblings have a two-fold greater concordance rate than half siblings [5]. These figures suggest that ASD heritability could be as great as 50 %. Potential transmission modes include copy number variation, single nucleotide variants, and single gene deletions. Nearly 2000 individual genes have been implicated in ASD [6], but none are specific to the disorder.

An alternative mechanism for ASD pathogenesis includes epigenetic regulation. Extracellular transport of miRNA (through exosomes and other microvesicles) is an established epigenetic mechanism by which cells can alter their own gene expression and the expression of genes in cells around them. For the latter to occur, vesicular miRNA is extruded into the extracellular space, docks and enters neighboring cells, and blocks translation of mRNA into proteins [7]. The extracellular nature of this process allows the measurement of genetic material from the central nervous system through simple collection of saliva [8]. This method minimizes many of the limitations associated with analysis of post-mortem brain tissue (e.g., anoxic brain injury, RNA degradation, post-mortem interval, agonal state), peripheral leukocytes (relevance of expression changes), or serum (painful blood draws) employed in previous studies [9–13]. Thus, extracellular miRNA quantification in saliva provides an attractive and minimally invasive technique for biomarker identification in children with ASD. The current study hypothesized that differential expression of brain-related miRNA may be detected in the saliva of ASD subjects, predictive of ASD classification, and related to neurodevelopmental measures of adaptive behavior.

## Methods

### Subjects and assessments

This study was approved by the Institutional Review Board for the Protection of Human Subjects (IRB) of the State University of New York (SUNY) at Upstate Medical University in Syracuse, New York. Subjects were recruited from the greater Syracuse area through the SUNY Upstate Pediatric and Adolescent Center and the SUNY Upstate Center for Development, Behavior, and Genetics. Exclusion criteria for both control and ASD subjects included an age less than 4 years or greater than 14 years, confounding neurological (i.e. cerebral palsy, epilepsy) or sensory (i.e. auditory or visual impairment) disorders, or acute illness. Wards of the state, subjects with mental retardation or a history of pre-term birth (less than 32 weeks gestation) or birth weight less than 10th percentile for gestational age were also excluded from participation. Subjects with a diagnosis of intellectual disability, ASD, or a family history of ASD were excluded from the control group. ASD subjects with a known syndromic phenotype (i.e. Rett Syndrome, Tuberous Sclerosis, Angelman Syndrome, Fragile X) were also excluded. Given the established comorbidity of psychiatric symptoms in children with ASD, subjects with attention deficit hyperactivity disorder (ADHD) or anxiety were not excluded.

Informed written parental consent and informed written subject assent (when possible) was obtained for a total of 45 subjects who were recruited for the study, including 24 subjects with a current diagnosis of ASD and 21 non-ASD control subjects (Table 1). ASD subjects were diagnosed according to DSM-5 (American Psychiatric Association, 2013) criteria and were evaluated with an age-appropriate module of the Autism Diagnostic Observation Schedule (ADOS), the Childhood Autism Rating Scale (CARS), and/or the Krug Asperger Index. The Vineland Adaptive Behavior Scales 2nd edition was administered to all children by a physician through parental interview to evaluate functional neurodevelopmental indices of communication, social interaction, and activities of daily living. Medical history, birth history, family history, surgical history, current medications, medical allergies, immunization status, and dietary modifications were obtained. A brief physical exam was performed to screen for neurologic deficits, visual/hearing impairment, or syndromic physical features.

**Table 1:** Subject characteristics

				Vineland adaptive behavior scales						
Controls	Age (years)	Sex	ADOS	Comm	Social	ADLs	Comp	Birth age (weeks)	Weight (%ile)	Height (%ile)
Mean	9.2	16M, 5F		110.1	104.4	100.4	105.3	39.2	78.2	68.4
StDev	2.5			10.0	15.7	11.0	12.7	1.3	16.5	20.6
Range	4–13			88–127	81–146	85–124	87–132	36–42	50–100	33–97
ASD										
Mean	9.1	19M, 5F	10.6	76.0	77.8	73.6	70.7	38.3	64.7	59.5
StDev	2.4		4.1	15.3	14.3	10.9	10.2	2.5	29.6	25.7
Range	5–13		3–16	49–113	47–108	52–95	48–90	31–41	5–99	10.99
*p* value	0.182	0.816		0.001	0.001	0.000	0.000	0.294	0.915	0.848

*ADLs* activities of daily living, *ADOS* autism diagnostic observation schedule, *Comm* Vineland Communication score, *Social* Vineland Socialization score, *Comp* Vineland Composite score. There were no differences in age or gender composition, birth age, weight or height. Note, the highly significant differences in Vineland scores between control and autism spectrum disorder (ASD) groups

There were no significant differences between groups in age (*p* = 0.18), sex (*p* = 0.82), weight (*p* = 0.91), height (*p* = 0.85), or birth age (*p* = 0.29). The mean age of the ASD subjects was 9.2 ± 2.5 years and the mean birth weight was 3.2 ± 0.64 kg. The ASD subjects had a mean ADOS score of 10.6 ± 4.1, consistent with DSM-5 criteria for mild to moderate ASD. Compared with control subjects they displayed significantly decreased levels of Communication (*p* <0.001), Social Interaction (*p* = 0.001) and Activities of Daily Living (*p* <0.001) as assessed by Vineland Adaptive Behavior Scales (Table 1).

Overall, the ASD group of 24 children included several with comorbid diagnoses: ADHD (*n* = 15), anxiety disorder (*n* = 8), learning disability or developmental delay (*n* = 5), asthma (*n* = 3), allergies (*n* = 2), obsessive-compulsive disorder (*n* = 2), and depression (*n* = 1). Reported medications in this group included: methylphenidate stimulants (*n* = 8), serotonin specific reuptake inhibitors (SSRIs; *n* = 7), guanfacine (*n* = 5), atypical antipsychotics (*n* = 5), clonidine (*n* = 1), bronchodilators (*n* = 3), anti-histamines (*n* = 3), multivitamins (*n* = 8) and omega-3 supplements (*n* = 4). Three of the probands were eating a modified gluten-free diet and no ASD subjects had any dental carries or periodontal disease. Five ASD subjects had a history of birth complications requiring neonatal intensive care, although none required care beyond 11 days. Most (*n* = 17) of the ASD subjects had a current or past history of educational intervention (speech therapy, physical therapy, occupational therapy). There were also several probands with positive family histories of neuropsychiatric and neurodevelopmental disorders (limited to 1st and 2nd degree relatives and 1st cousins): learning disability (*n* = 10), depression (*n* = 8), anxiety disorder (*n* = 7), ADHD (*n* = 6), ASD (*n* = 4), and bipolar disorder (*n* = 3).

The control group of 21 typically developing children also included several with comorbid diagnoses: ADHD or ADD (*n* = 5), asthma (*n* = 6), eczema (*n* = 4), and allergies (*n* = 2). Reported medications in the control group included: methylphenidate (*n* = 3), bronchodilators (*n* = 6), and antihistamines (*n* = 5). None of the control children were eating a modified or gluten-free diet and one subject had dental carries. One control subject had a history of birth complication (RSV infection) that required a brief period of neonatal care. Three of the control subjects had a current or past history of educational intervention (speech therapy, physical therapy, occupational therapy). Positive family histories among 1st and 2nd degree relatives and 1st cousins were identified for learning disability (*n* = 2), depression (*n* = 1), ADHD (*n* = 1), and bipolar disorder (*n* = 1).

### Saliva collection and miRNA processing

Subjects were recruited during well-child visits. Saliva samples were collected in a non-fasting state between 10 am and 3 pm. After rinsing with tap water, approximately 3 mLs of saliva were obtained via expectoration using an Oragene RNA collection kit (DNA Genotek; Ottawa, Canada) and stored at room temperature until processing by the SUNY Molecular Analysis Core at Upstate Medical University. Salivary miRNA was purified using a standard Trizol method, followed by a second round of purification using the RNeasy mini column (Qiagen). The yield and quality of the RNA samples was assessed using the Agilent Bioanalyzer prior to library construction using the Illumina TruSeq Small RNA Sample Prep protocol (Illumina; San Diego, California). Multiplexed samples were run on an Illumina MiSeq instrument using v3 reagents at a targeted depth of 3 million reads per sample. Reads were aligned to the hg19/GRC37 build of the human genome in Illumina BaseSpace Software using the Bowtie algorithm in the MSR: Small RNA application (version: 1.0.0) and normalized to reads per million (RPM) prior to analysis. The data set supporting the results of this article is available in the NCBI Sequence Read Archive (BioProject Accession: PRJNA310758; BioSample Submission ID: SUB1330937).

### Statistical analysis

Analysis of the combined medical, demographic, and neuropsychological data was performed to identify significant group differences between ASD and control subjects. Individual miRNAs were used for comparisons between groups only if they were detected in at least half the samples regardless of diagnosis. A total of 246 miRNAs were tested. Because the RNA-Seq data were not normally distributed, group differences in miRNA levels were examined using a non-parametric Wilcoxon Mann-Whitney U test with Benjamini-Hochberg False Discovery Rate (FDR) correction for multiple comparisons. The miRNAs with FDR values <0.15 were initially used in individual logistic regression analyses to assess discriminative power in an idealized “best-fit” approach. The rationale for doing so was the fact that logistic regression makes no assumption about the distribution of the original RNA-Seq data and it is highly effective at iteratively determining an optimal model for the data using the logistic function *Y* = [1/(1 + e^-(a + b1X1 + b2X2 + bnXn + …)^)] that best describes the dependency of the dependent outcome (diagnosis, coded as 0 or 1) on the full set of 14 independent variables. This best fitting is accomplished by adjustment of the partial regression coefficients for each miRNA variable until an optimal solution is obtained using the Maximum Likelihood criterion. During this process, each subject sample is determined to have a specific likelihood of falling in one of the diagnostic classes based on the model and the total likelihood (L) for the set of subjects is derived from the running product of the likelihood scores for all of the subjects. Since a prediction is made for each subject, the results of the logistic regression analysis are then used to produce a 2 × 2 classification table from which we can determine the Sensitivity or True Positive Rate (i.e., fraction of ASD subjects who were correctly predicted to be ASD based on the model) and the Specificity or True Negative Rate (i.e., the fraction of Control subjects who were correctly predicted to be Controls). The cutoff points for the classification were set by default to be *Y* = 0.5 (halfway between the diagnostic category coding of 0 and 1). By varying the cutoff point across the full range of cutoff values and recalculating the Sensitivity and Specificity at each point, it is then possible to construct a Receiver Operating Characteristic (ROC) curve which provides an unbiased assessment of the overall model performance.

To facilitate comparisons with other data sets, mean differences in abundance seen in ASD subjects were reported as normalized Z score differences relative to controls as well as standardized Cohen’s d values, which incorporate the variability within each subject group. We also reported the Wald statistics with resulting p values for each of the individual regression results. Comparisons of miRNA levels to various medical, demographic and neuropsychological measures were performed using Spearman’s rank correlation.

One of the limitations of any regression modeling approach is the possibility that the “best fit” only accurately predicts outcomes in the initial (discovery) data set. To more stringently evaluate the empirical validity of the 14 miRNAs, we performed classification testing and ROC curve analysis based on the results of 100-fold Monte-Carlo Cross Validation (MCCV) with balanced subsampling. In each iteration two-thirds of the samples were used to evaluate the miRNA feature importance. Next, the 2, 3, 5, 7, 10 and 14 most important classifying miRNA features were used to build classification models which were cross-validated on the remaining one-third of the samples that were left out. This was repeated 100 times to determine the performance and confidence interval of each model. To further complement the logistic modeling we did in the discovery phase, this MCCV analysis was performed using the multivariate linear regression approach of Partial Least Squares Discriminant Analysis (PLS-DA). This method extracts multidimensional linear combinations of the 14 miRNA features that best predict the class membership or diagnosis (*Y*). These analyses were performed using the Metaboanalyst 3.0 server which implements the plsr function provided by the R *pls* package, with classification and cross-validation performed using the *caret* package. We also used this tool to rank the variables by their relative importance, as determined by the sum of regression coefficients in the different simulated models, and generate individual boxplots for the 4 most robust differentially expressed miRNAs.

To visualize the expression patterns and general separation power of the set of significantly changed miRNAs, we then used hierarchical clustering with a Euclidian distance metric to group miRNAs with similar patterns together, and visualized the subjects in the three eigenvector dimensions created from the PLS-DA analysis of the 14 miRNAs.

Systems-level analysis of the miRNA data was performed using the miRNA Data Base (miRDB) online resource to provide the predicted targets for each of the mature sequences that we identified (according to mirBase v21 August 2014 annotation). This database version identifies 2588 human miRNAs and 947,941 target interactions. The interactions that were revealed were then filtered based on the predicted strength of the miRNA-mRNA interaction, as reflected in the miRDB output, to include only the top 20 % of predicted targets for each miRNA. These specific mRNAs were then examined for evidence of functional enrichment using the online Functional Annotation Clustering tool from the Database for Annotation, Visualization, and Integrated Discovery (DAVID, version 6.7) at the National Institute of Allergy and Infectious Diseases (NIAID). Because of the large number of genes being examined, we increased the EASE score threshold to 2.0, and set the Multiple Linkage Threshold to 0.7, the Similarity Threshold to 0.45, and the Final Group Membership size to 4. Only the top three Annotation Clusters were reported in table form. In addition to the DAVID functional clusters, we also compared the list of the top 20 % of predicted targets for the combined set of 14 miRNAs to the 740 ASD-associated genes catalogued in the Simons Foundation Autism Database (AutDB) and tested for possible enrichment using a Fisher’s Exact test and Odds Ratio calculation.

Brain and tissue-specific expression patterns for differentially expressed miRNAs were identified by review of a survey of differentially expressed miRNAs across the developing and adult human brain [14, 15]. We also used the brain data to note whether miRNAs that were highly-expressed in brain were also detected in the saliva regardless of whether they were altered in ASD.

## Results

### Saliva miRNA levels show relationship to diagnosis and adaptive behavior measures

Sequencing of salivary miRNAs detected 246 miRNAs as being present in at least half the samples. Among these, 14 miRNAs showed significant changes in expression according to a Mann-Whitney test (*p* <0.05, FDR <0.15) in the ASD group compared with controls (Table 2). Ten of the miRNAs were up-regulated in ASD subjects and four were down-regulated. The miRNA with the largest mean difference in abundance between ASD and control subjects was miR-628-5p (it also had the most significant difference) (*p* = 0.0001, Z score difference = 1.13). Results from the individual logistic regression analyses also highlighted miR-628-5p as the most significant (Wald statistic = 11.21, *p* = 0.001), and it showed the second highest area under the curve (AUC = 0.90) from the ROC analysis (Table 2). Individually, miR-335-3p had the largest AUC and miR-30e-5p had the highest accuracy in predicting ASD diagnosis at 76 % (Table 2).

**Table 2:** Top-ranked variables distinguishing ASD from Control subjects and their correlations with neurodevelopmental measures

Group Mean/Median comparisons					Logistic regression classifications				Neurodevelopmental correlations									
miRNA	M-W *-val*	FDR	Z diff	Cohen’s d	Wald	*p*-Val	AUC	Accuracy	age(Yrs)	ADOS comm	ADOS social	ADOS C+S	VABS comm	VABS ADL	VABS social	VABS comp	Sequence	miRBase ID
miR-628-5p	0.0001	0.027	1.13	0.83	11.21	0.001	0.90	0.73	−0.260	−0.037	−0.286	−0.210	**−0.346**	**−0.354**	**−0.381**	**−0.399**	AUGCUGACAUAUUUACUAGAGG	MIMAT0004809
miR-127-3p	0.003	0.040	0.62	0.96	2.55	0.110	0.86	0.64	−0.347	−0.249	−0.189	−0.149	**−0.363**	**−0.414**	**−0.463**	**−0.453**	UCGGAUCCGUCUGAGCUUGGCU	MIMAT0000446
miR-27a-3p	0.0013	0.110	−0.089	0.90	7.00	0.008	0.78	0.71	0.141	−0.036	−0.028	−0.035	**0.353**	**0.357**	**0.414**	**0.418**	UUCACAGUGGCUAAGUUCCGC	MIMAT0000084
miR-335-3p	0.0014	0.089	0.95	0.89	7.40	0.007	0.92	0.73	−0.199	0.247	−0.075	0.019	**−0.462**	**−0.512**	**−0.492**	**−0.505**	UUUUUCAUUAUUGCUCCUGACC	MIMAT0004703
miR-2467-5p-	0.0015	0.074	0.87	0.91	7.11	0.008	0.82	0.73	−0.020	−0.138	0.003	−0.003	**−0.381**	**−0.365**	**−0.368**	**−0.399**	UGAGGCUCUGUUAGCCUUGGCUC	MIMAT0019952
miR-30e-5p	0.0017	0.069	−0.90	0.90	7.52	0.006	0.77	0.76	0.191	0.076	−0.278	−0.260	**0.368**	**0.496**	**0.499**	**0.496**	UGUAAACAUCCUUGACUGGAAG	MIMAT0000692
miR-28-5p	0.0021	0.072	0.90	0.90	8.25	0.004	0.81	0.69	0.190	0.054	0.379	0.332	**−0.405**	**−0.422**	**−0.420**	**−0.431**	AAGGAGCUCACAGUCUAUUGAG	MIMAT0000085
miR-191-5p	0.0029	0.089	0.94	0.89	7.97	0.005	0.76	0.69	−0.171	0.336	0.221	0.337	−0.267	−0.206	−0.291	**−0.299**	CAACGGAAUCCCAAAAGCAGCUG	MIMAT0000440
miR-23-3p	0.0031	0.085	−0.90	0.90	7.63	0.006	0.76	0.69	0.151	−0.115	−0.268	−0.223	**0.421**	**0.489**	**0.460**	**0.487**	AUCACAUUGCCAGGAUUUCC	MIMAT0000078
miR-3529-5p	0.0033	0.082	0.80	0.93	6.80	0.009	0.76	0.64	−0.091	0.325	0.230	0.290	**−0.458**	**−0.353**	**−0.466**	**−0.462**	AACAACAAAAUCACUAGUCUUCCA	MIMAT0022741
miR-218-5p	0.0035	0.077	0.59	0.96	3.43	0.064	0.79	0.73	0.045	−0.059	0.061	0.058	−0.246	−0.261	**−0.314**	−0.296	UUGUGCUUGAUCUAACCAUGU	MIMAT0000275
miR-7-5p	0.0045	0.091	0.59	0.97	3.19	0.074	0.86	0.73	0.007	−0.095	0.143	0.090	**−0.405**	**−0.389**	**−0.414**	**−0.447**	UGGAAGACUAGUGAUUUUGUUGU	MIMAT0000252
miR-32-5p	0.0051	0.097	−0.86	0.91	7.07	0.008	0.75	0.73	0.238	0.269	0.146	0.139	0.297	**0.358**	**0.386**	**0.361**	UAUUGCACAUUUACUAAGUUGCA	MIMAT0000090
miR-140-3p	0.0078	0.137	0.64	0.96	4.25	0.039	0.84	0.73	−0.046	−0.200	−0.163	−0.241	−0.152	−0.243	−0.217	−0.233	UACCACAGGGUAGAACCACGG	MIMAT0004597

Abbreviation: *AUC* area under the curve, *FDR* false discovery rate, *C+S* Communication + Socialization, *M-W p-val* Mann-Whitney *p*-value, *VABS* Vineland Adaptive Behavior Scales, *Wald* Wald statistic

Note that overall, the 14 miRNAs listed were 91% accurate, although accuracy for individual miRNAs did not exceed 0.76. Correlations shown in bold were significant (*p* <0.05). Also note that several in RNAs showed robust correlations with Vineland scores

To determine if the 14 miRNAs of interest were associated with neurodevelopmental measures, we performed Spearman’s rank correlation analysis. This analysis revealed significant correlations between multiple measures included in the Vineland scores and 13 of the 14 miRNAs (only miR-140-3p failed to show significant correlations). Notably, nine of the miRNAs demonstrated only negative correlations (i.e., higher miRNA was associated with lower Vineland scores) while four of them showed only positive correlations. Furthermore, every miRNA with positive correlations to Vineland scores was one that had reduced expression in ASD subjects, whereas every miRNA with negative correlations to Vineland scores had increased expression in ASD (Table 2).

### Hierarchical clustering and linear discriminant analysis distinguish samples by miRNA levels

**Fig. 1:**
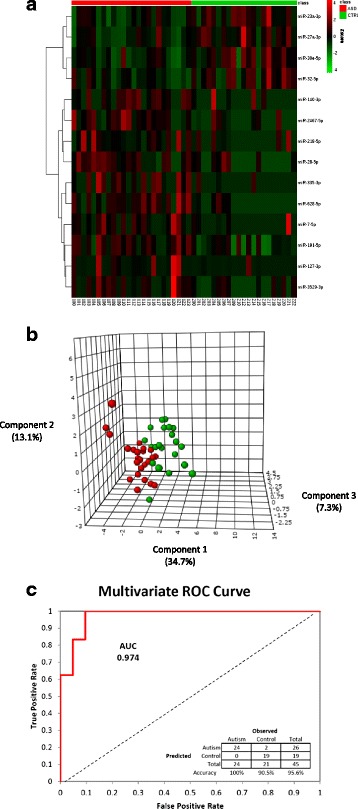
Differential expression and diagnostic utility of miRNAs in saliva of ASD children. **a** Hierarchical cluster analysis of the top 14 miRNAs. These miRNAs were differentially expressed in ASD children compared with Controls. Color indicates average Z-score of normalized abundance for each gene. A Euclidian distance metric was used with average cluster linkages for this figure. **b** Partial Least Squares Discriminant Analysis (PLS-DA) of the top 14 miRNAs showed the general separation of subjects into two clusters, using only three eigenvector components (x, y, and z axes labeled Component 1, Component 2, and Component 3) that collectively accounted for 55 % of the variance of the data set. **c** ROC-AUC analysis of the training data set indicated a very high level of performance in the logistic regression classification test (100 % sensitivity, 90 % specificity, with an AUC of 0.97)

Hierarchical clustering was performed for ASD and Control subjects to reveal salient patterns in the miRNA data (Fig. 1a). The PLS-DA results were used to visualize the degree of separation between ASD and Control subjects using a three-dimensional representation of the 14 variable matrix. The results of this analysis complemented the clustering results and indicated only moderate overlap in the subject groups (Fig. 1b). Examination of the medical, demographic, and adaptive behavior data for these overlapping Control and ASD subjects in the 3-dimensional plots failed to identify any definitive explanations.

**Fig. 2:**
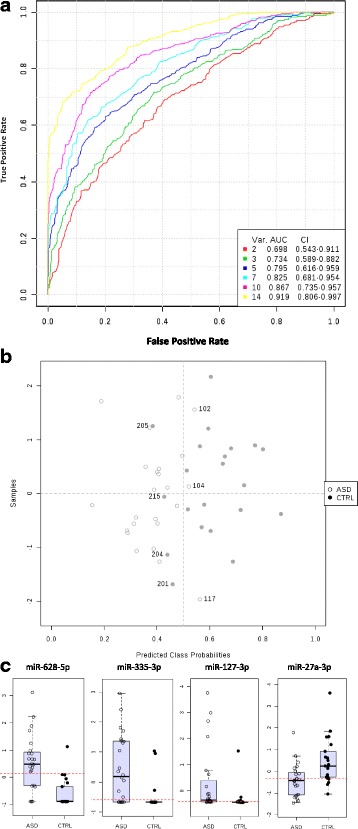
Monte-Carlo Cross-Validation analysis of the top 14 miRNAs. **a** The robustness of the 14 miRNA biomarkers was evaluated in stepwise fashion by determining their ability to correctly classify subjects using 100 iterations of a multivariate PLS-DA with 2, 3, 5, 7, 10, and 14 miRNAs included, and masking of 1/3 of the subjects during the training phase. This revealed an overall ROC-AUC of 0.92 and mis-classification of three ASD and four Control subjects. **b** Shows the classification of subjects plotted by predicted class probabilities from the MCCV (x axis), with incorrectly classified subjects identified by ID number. The y axis units are arbitrary. **c** Whisker box plots (showing median and inter-quartile range) of the four most robustly changed miRNAs according to the Mann-Whitney test

### Multivariate regression, class prediction and ROC analysis indicate high sensitivity and specificity

The initial “best-fit” model to assess the maximal diagnostic utility was based on a single multivariate logistic regression test for classification accuracy. The results of this were evaluated using a ROC curve and classification prediction table (Fig. 1c). The multivariate ROC plot for this set of miRNAs revealed an area under the curve (AUC) of 0.974. This miRNA set was 100 % sensitive and 95.6 % specific for predicting the diagnosis of ASD within the study participants. Notably, because we pre-selected our subjects into either ASD or control groups, we did not determine the Positive Predictive Value or Negative Predictive Value of the 14 variables.

### 100-fold cross-validation of diagnostic utility of miRNA data Set

Our data set of 14 miRNAs variables continued to perform at a very high level in the Monte-Carlo Cross-Validation (MCCV) analysis (Fig. 2a), with an average ROC AUC value of 0.92 for the full model (containing all 14 miRNAs). Furthermore, the MCCV revealed 87.5 % specificity and 81 % sensitivity, with an overall accuracy of 84.4 % across 100 simulations. The most common outcome was a confusion matrix that contained four misclassified Controls and three or four misclassified ASD subjects. Notably, these were the same subjects that were misclassified using our original logistic regression method, suggesting either a linear or non-linear multivariate modeling approach is appropriate.

### Pathway enrichment analysis identifies enrichment for neurodevelopment and ASD targets

Analysis of the highest-confidence target mRNAs of the 14 miRNAs yielded an average of 555 predicted targets for each miRNA (7764 total predicted interactions). Approximately 10 % of these were targeted by more than one of the miRNAs, yielding approximately 7000 distinct mRNA interactions (Additional file 1: Table S1). This large number of interactions was then filtered based on the predicted strength of the miRNA-mRNA interaction, as reflected in the miRDB output to include only the top 20 % of predicted targets for each miRNA (Additional file 1: Table S1, italicized genes). This identified 1347 unique strongly-predicted mRNA interactions. The specific high-confidence mRNAs were then examined for evidence of functional enrichment using DAVID (version 6.7). A total of 1247 of the high-confidence mRNA targets had functional annotation available. Using stringent settings (EASE score threshold set to 2.0, with Multiple Linkage Threshold set to 0.7, Similarity Threshold set to 0.45, and Final Group Membership set to four) revealed 310 total cluster mappings. The top subnodes in the annotation clusters were then examined, revealing more than 2-fold enrichment of genes involved in Transcriptional Activation (present in five subnodes and two annotation clusters) and genes involved in Neuron Projection (51 genes) and Axon Projection (31 of the same genes).

We further probed the high-confidence target genes for relevance to ASD by comparing them with the 740 protein-coding genes in the Simons Foundation Autism Database (AutDB). Our high-confidence list of 1347 mRNA targets contained 108 (14.6 %) that overlapped the AutDB list (Additional file 2: Table S2) [16]. This represented a significant 2.2-fold enrichment for ASD-associated genes compared to that expected by chance alone (Odds Ratio = 2.40, 95th CI = 1.94–2.97, Fisher’s Exact *p* value = 7.1e-12). Notable among these ASD-associated mRNA targets were Fragile X Mental Retardation (FMR1) and Forkhead Box Protein P2 (FOXP2).

The genes which mapped to the enriched DAVID clusters and the AutDB candidate genes were combined to indicate those target mRNAs that might be expected to have the most functional relevance for ASD. This indicated the most enhancement for AutDB genes that mapped to the Neuron Projection and Axon Projection subnodes, and also highlighted a small number of genes with apparent pleiotropic effects on multiple subnodes, including FOXP2 and FMR1 (Additional file 3: Table S3).

### Target miRNAs in the saliva are widely and highly expressed in human brain

As a final examination of the potential brain-relevance of the miRNA targets we identified, we analyzed RNA sequencing data on miRNAs across the developing human brain (4 months to 23 years) as deposited in the public domain by Ziats and Rennert [14]. This analysis yielded results for 13 of the 14 miRNAs we found altered in ASD saliva. Notably, all 13 miRNAs were expressed in multiple brain regions, including the cerebellum, dorsolateral prefrontal cortex (PFC), ventrolateral PFC, medial PFC, orbitofrontal PFC, and hippocampus throughout childhood. Moreover, nine of the miRNAs were expressed at high read levels (>1000) while four were expressed at relatively low read levels (<1000). No relationship was seen between expression level and either age or sex. However, five of the miRNAs varied in expression across brain regions (miR-7-5p, miR-27a-3p, miR-140-3p, miR-191-5p, and miR-2467-5p) with most differences occurring in the cerebellum versus the hippocampus.

## Discussion

Current screening methods for ASD rely on parental questionnaires that are less than 50 % specific and not valid until 18–24 months of age. This approach is inefficient in identifying children with ASD and enrolling them in early intervention services [17]. The results of the present study suggest that addition of saliva-based biomarker testing could potentially lower the age of diagnosis and improve the specificity of screening, reducing the burden on referral services. We suggest that ideal biomarker candidates should be 1) expressed in the brain, 2) physiologically or functionally relevant to neurodevelopment, 3) easily measured from peripheral samples, and 4) differentially expressed in individuals with ASD. This study has identified a set of 14 miRNAs in the saliva that fit these criteria.

Examination of miRNA expression patterns across human brain development demonstrated that the miRNAs within our set of 14 biomarker candidates were consistently expressed in multiple brain areas throughout childhood. Moreover, functional pathway analysis of these miRNAs revealed enrichment of gene networks involved in neurodevelopment as well as genes associated with ASD according to the Simons Foundation Autism Database. Although it is beyond the scope of this report to discuss all of the overlapping target mRNAs, we do point out two notable ones: Fragile X Mental Retardation 1 (FMR1) and Forkhead Box Protein P2 (FOXP2). The FMR1 protein product is widely expressed in neurons [18], regulates synaptic translation through miRNA interactions [19], and is disrupted in Fragile X Syndrome, the most common inherited cause of intellectual disability. Approximately 40 % of children with Fragile X Syndrome meet the criteria for ASD. In a similar fashion, FOXP2 was the first gene implicated in developmental speech and language disorders [20], and missense mutations of FOXP2 result in verbal apraxia, a hallmark of ASD. Both of these genes were present in more than one subnode, with FOXP2 highly represented in both the Transcriptional Activation and Neuron Projection subnodes.

Of the 246 miRNA targets measured in the saliva of ASD and Control children, a set of 14 showed significant differences in abundance and was more than 95 % accurate at predicting ASD diagnosis in a multivariate nonlinear logistic regression model developed in the full discovery data set. 100-fold cross-validation using Monte-Carlo simulations with masking of 1/3 of the samples revealed 87.5 % specificity and 81 % sensitivity, with an overall accuracy of 84.4 %. Together, these findings indicate that miRNA profiling of the saliva has the potential to nearly double the overall specificity of the current “gold standard” M-CHAT-R screening method.

In the training set, all of the ASD subjects were correctly classified and only two control subjects were misclassified (subjects 204 and 205). However, these subjects did not display extreme variation in Vineland scores (both had composite scores of 113) or age (8 and 11 years old), although one subject did have a past history of speech therapy (subject 205). Their only notable medical findings were a diagnosis of asthma treated with albuterol as needed (subject 204) and the fact that both children were heavier and taller on average than the age-based percentiles of the children in the control group (weight percentiles 96 and 100 compared with a group mean of 78; height percentiles 92 and 97 compared with a group mean of 71). Examination of the records of the additional children who were misclassified during the cross-validation also failed to reveal any consistent pattern or association of medical or demographic variables.

A number of the salivary miRNAs that we identified as differentially expressed in children with ASD have been previously described in studies of post-mortem cerebellar cortex (miR-23a-3p, miR-27a-3p, miR-7-5p, and miR-140-3p) [9], lymphoblastoid cell lines (miR-23a-3p, miR-30e-5p, miR-191-5p) [10–12], and serum (miR-27a-3p, miR-30e-5p) [14] of children with ASD. Thus, there are three miRNAs differentially regulated across three tissue types in children with ASD (miR-23a-3p, miR-27a-3p, and miR30e-5p). It is worth noting that miR-23a functions cooperatively with miR-27a to regulate cell proliferation and differentiation [21] and the pair of miRNAs have been reported to be dysregulated in a number of human disease states, including ASD [11, 22]. Levels of miR-23a also fluctuate in response to CNS injuries such as cerebral ischemia [23] or temporal epilepsy [24], both of which can be associated with ASD [18]. Thus, the dysregulation of miR-23a-3p may represent a pathophysiological hallmark of ASD.

The most robustly altered miRNA (miR-628-5p) in the present study has not been identified in previous ASD studies, although it is expressed in the human brain throughout postnatal development [14] and has been implicated in CNS pathology [25]. For example, analysis of miRNA expression in human gliomas showed significantly decreased expression of miR-628-5p [25]. This contrasts with miR-628-5p expression in the saliva of ASD subjects, where it was significantly increased.

Aside from the sample size and cross-sectional nature of this pilot study, another limitation is the age of ASD and control subjects it describes (4–14 years) which are not representative of the target population in which ASD biomarkers would ideally be utilized (0–2 years). However, selecting a homogenous group of subjects with mild ASD (as measured by ADOS) that was well-established and diagnosed by a developmental specialist requires subjects with long-standing diagnoses. An additional consideration is the feasibility of saliva collection for screening children less than two years of age. We suggest that saliva is not only found in abundance during the period of teething (6 months to 18 months), but is also the most painless to collect. Future studies will be needed to assess the utility of the current miRNAs in predicting outcomes based on saliva samples from children in this age range.

## Conclusions

The novel aspect of this study is that it identifies a set of miRNAs in the saliva that are expressed in the brain, impact genes related to brain development and ASD, and are changed in a highly-specific manner in children with ASD. The specificity of this set of 14 miRNAs for a diagnosis of ASD is nearly twice that of the M-CHAT-R, the current gold standard used in ASD screening. Though copy number variants (CNVs) and single nucleotide polymorphisms (SNPs) are considered important genetic risk factors for ASD [26], they account for less than 30 % of cases when considered in total [27] and no single CNV explains more than 1 % of ASD incidence [27, 28]. In comparison, epigenetic mechanisms such as miRNAs have the potential to alter coordinated networks of genes related to specific functional classes. An unexpected finding in the present study was the relationship of saliva miRNA levels with standard neurodevelopmental measures of adaptive behavior and the convergence of miRNAs targets on both neurodevelopmental processes and ASD candidate genes. This makes miRNAs such as miR-27a, miR-23a and miR-628-5p intriguing potential functional biomarkers for ASD. Although the results in the present study must be viewed as preliminary in nature, prospectively validating the miRNA changes in a population of younger children with positive M-CHAT-R questionnaires and larger independent cohort replication samples could provide compelling evidence for the addition of miRNA biomarker screening to the diagnosis of ASD.

### Availability of supporting data

Supplementary Tables accompany this article. Sequencing data set are available in the NCBI Sequence Read Archive (BioProject Accession: PRJNA310758; BioSample ID: SUB1330937).

## Additional files

Additional file 1: Table S1. All mRNA targets of 14 miRNA biomarkers (20 % highest-confidence targets for each miRNA are italicized, and their number is listed at the top of each column). (XLSX 116 kb) Additional file 2: Table S2. High confidence AutDB genes targeted by one or more of the 14 candidate biomarker miRNAs. (XLSX 18 kb) Additional file 3: Table S3. Top enriched functional clusters revealed by functional annotation clustering of high confidence mRNA target genes of candidate miRNA biomarkers. (XLSX 32 kb)

## Abbreviations

ADHD: attention deficit hyperactivity disorder
ASD: autism spectrum disorder
AUC: area under the curve
FDR: false discovery rate
FMR1: fragile X mental retardation protein 1
FOXP2: forkhead box protein 2
MCCV: Monte-Carlo Cross Validation
miRNA: micro ribonucleic acid
RNA-Seq: RNA-sequencing
ROC: receiver operating characteristic
VABS: Vineland Adaptive Behavior Scales
